# Chasing the FOXO3: Insights into Its New Mitochondrial Lair in Colorectal Cancer Landscape

**DOI:** 10.3390/cancers11030414

**Published:** 2019-03-23

**Authors:** Valentina Grossi, Candida Fasano, Valentina Celestini, Martina Lepore Signorile, Paola Sanese, Cristiano Simone

**Affiliations:** 1 Medical Genetics, National Institute for Gastroenterology, IRCCS ‘S. de Bellis’, Via Turi, 27, Castellana Grotte, 70013 Bari, Italy; candida.fasano@irccsdebellis.it (C.F.); leporesignorile.labsimone@gmail.com (M.L.S.); 2 Division of Medical Genetics, Department of Biomedical Sciences and Human Oncology (DIMO), University of Bari Aldo Moro, Piazza G. Cesare, 11, 70124 Bari, Italy; celestini.labsimone@gmail.com (V.C.); sanese.labsimone@gmail.com (P.S.); 3 Department of Molecular Medicine, Sapienza University of Rome, Viale Regina Elena, 324, 00161 Roma, Italy

**Keywords:** colorectal cancer, chemoresistance, post-translational modifications, mitochondrial FOXO3a, cancer-related signaling pathways, precision cancer medicine, metformin

## Abstract

Colorectal cancer (CRC) poses a formidable challenge in terms of molecular heterogeneity, as it involves a variety of cancer-related pathways and molecular changes unique to an individual’s tumor. On the other hand, recent advances in DNA sequencing technologies provide an unprecedented capacity to comprehensively identify the genetic alterations resulting in tumorigenesis, raising the hope that new therapeutic approaches based on molecularly targeted drugs may prevent the occurrence of chemoresistance. Regulation of the transcription factor FOXO3a in response to extracellular cues plays a fundamental role in cellular homeostasis, being part of the molecular machinery that drives cells towards survival or death. Indeed, FOXO3a is controlled by a range of external stimuli, which not only influence its transcriptional activity, but also affect its subcellular localization. These regulation mechanisms are mediated by cancer-related signaling pathways that eventually drive changes in FOXO3a post-translational modifications (e.g., phosphorylation). Recent results showed that FOXO3a is imported into the mitochondria in tumor cells and tissues subjected to metabolic stress and cancer therapeutics, where it induces expression of the mitochondrial genome to support mitochondrial metabolism and cell survival. The current review discusses the potential clinical relevance of multidrug therapies that drive cancer cell fate by regulating critical pathways converging on FOXO3a.

## 1. Introduction 

Colorectal cancer (CRC) is the second leading cause of death from tumor in the Western world [1]. The vast knowledge gained over the past twenty years has provided significant clues about the genes and pathways leading to CRC, indicating that it is not one disease, but rather a collection of molecularly distinct neoplastic disorders. Progression of CRC is usually the result of sequential alterations in signaling pathways such as WNT, PI3K, EGFR, p53, and TGFβ [2,3,4,5]. 

Furthermore, a new list of cancer driver genes has been unveiled by large-scale studies taking into account the high degree of inter-tumor heterogeneity [6,7,8,9,10]. The Cancer Genome Atlas (TCGA) network has characterized the genomic features of human tumors [11,12,13,14,15] and described how genomic alterations drive cancer [10]. The TCGA study on CRC collected an integrative analysis of genomic data to provide further insights into the pathways that are dysregulated in CRC. In addition to the expected APC, p53, SMAD4, PIK3CA, and KRAS mutations, new findings included frequent mutations in ARID1A, SOX9, and FAM123B, suggesting an important role for WNT pathway and Myc-directed transcriptional activation and repression [15]. Furthermore, to facilitate clinical translation, the CRC Subtyping Consortium (an international consortium dedicated to large-scale data sharing and analytics) described a new taxonomy of the disease based on the results from six independent classification systems [16]. Their *in silico* strategy includes the development of a Cancer Drivers Database, containing lists of genes that drive tumorigenesis, and a Cancer Drivers Actionability Database, containing a comprehensive set of current anticancer targeted drugs and sets of rules to prescribe them to patients [16]. 

Precision medicine is defined as the process of matching an individual patient with the medicines that are best for them; however, some investigators have expressed concerns about its use in cancer [17]. Recent studies have shown that most cancer patients who undergo genomic testing do not benefit from this strategy [18,19,20,21,22,23]. As a matter of fact, only a few types of cancer have been successfully treated with a single drug, e.g., imatinib in chronic myeloid leukemia [24], gefitinib (EGFR inhibitor) in mutant EGFR non-small cell lung cancer, and PLX4032 (BRAF inhibitor) in mutant BRAF melanoma [25,26,27,28]. On the whole, genome-driven therapies have helped a minority of patients with advanced cancer [29]. Importantly, the occurrence of chemoresistance is responsible for the limited success of various drugs, leading many patients to relapse after a couple of years of treatment. Indeed, blocking one pathway, such as RAS, is likely to induce only a cytostatic effect, while inhibiting a crosstalk resistance pathway is expected to promote chemosensitivity and a final cytotoxic effect [30]. In this context, combined therapies might prove beneficial to improve patient outcomes. 

New therapeutic approaches may benefit from molecular profiling and preclinical investigation aimed at identifying specific drugs targeted against the crucial drivers of CRC pathogenetic pathways. In addition to the expected APC, p53, SMAD4, PIK3CA, and KRAS mutations, significant genomic aberrations have been found in IGF2, IGFR, ERBB2, ERBB3, MEK, AKT, mTOR, and SRC, which all converge on the FOXO3a transcription factor. FOXO3a is involved in a wide range of cellular processes and is a key determinant of cancer cell homeostasis, playing a dual role in the survival/death response to metabolic stress and cancer therapeutics [31]. These findings suggest that several proteins in critical cancer-related signaling pathways, such as the RTK-RAS and PI3K cascades, might be targeted for inhibition [15]. As a general principle, since direct inhibition of the target is often not sufficient, better results are likely to be obtained by addressing the upstream signaling networks. Based on the assumption that crosstalk pathways may play a compensatory role in CRC progression, in this review we will discuss the therapeutic potential of inhibitor cocktails. In addition to taking an unbiased look at novel associations that might affect CRC viability by sensitizing cells to chemotherapy through modulation of mitochondrial FOXO3a-dependent chemoresistance, we will provide insight into genes and pathways involved in tumor resistance to support the development of more tailored interventions in CRC patients. 

## 2. The FOXO Family and FOXO3a 

The forkhead box (FOX) protein family includes more than 100 transcription factors, which can be grouped into 19 subclasses sharing a conserved DNA-binding domain, a sequence of 80 to 100 amino acids called the forkhead domain. FOX transcription factors are involved in diverse functions, including development, metabolism, tumorigenesis and cognition, and their role is regulated through the interaction with a variety of binding partners, including co-activators, co-repressors and other transcription factors [32]. FOXO proteins have been identified in nematodes (*Caenorhabditis elegans*), zebrafish (*Danio rerio*), fruit flies (*Drosophila melanogaster*), mice, and humans [33,34]. Mammalian FOXOs are orthologues of the transcription factor DAF-16 identified in *C. elegans*, which is involved in insulin signaling and longevity [35]. The FOXO class comprises four members, FOXO1, FOXO3a, FOXO4, and FOXO6, sharing high sequence homology. Additionally, it has been recently unveiled that the human FOXO3a pseudogene FOXO3b is a true human gene that encodes a FOXO3a-related protein product [36]. The structure of FOXO proteins consists of four common main domains: the forkhead DNA binding domain (DBD), a nuclear localization signal (NLS) downstream of the DBD, a nuclear export sequence (NES) and a C- terminal transactivation domain [37]. Through their DBD, FOXO proteins bind to the consensus motif (forkhead response element, FHRE) 5’-TTGTTTAC-3’ within the target promoter sequence [38]. Once they are bound to DNA, their C-terminal transactivation domain initiates gene transcription, acting as a transcriptional activator or repressor depending on the range of associated co-factors that are recruited. 

Most FOXO proteins are ubiquitously expressed but not equally distributed in all tissues, suggesting that individual members may have specificity with regards to cellular function [39]. In particular, FOXO1 transcript is present at high levels in adipose tissues, FOXO4 is mostly expressed in skeletal muscle, FOXO3a transcript is abundant in the brain, heart, kidney and spleen, while FOXO6 is predominantly found in the brain. Through their transcription factor activity, FOXO proteins differentially affect cell fate in a downstream target-specific manner, showing a great diversity in function as a result of their ability to bind to a large number of gene promoters containing the DBD [39]. 

In invertebrates, the FOXO homologue DAF-16 has been proven to increase lifespan and regulate nutrient sensing. These functions are conserved in Drosophila, where FoxO is also involved in insulin signaling. In mammals, FOXOs play a role in stress resistance, proliferation/arrest, survival/death, metabolism, and autophagy; moreover, they are involved in tumor suppression, regulation of energy metabolism, and development in specific tissues [40]. One of the FOXO proteins’ most explored functions is the regulation of the cell cycle at various stress-response checkpoints. Indeed, they can block G1 progression by repressing cyclins D1 and D2 either directly [40] or indirectly through Bcl6/STAT5 [41]. FOXOs also promote cell cycle arrest at the G1/S phase by upregulating the cyclin-dependent kinase (CDK) inhibitors p21 [42] and p27 [43]. Furthermore, FOXO3a can act on p27 directly by disrupting cyclin D/CDK4 and cyclin E/CDK2 complexes [43], or indirectly through the modulation activity exerted by c-Myc on FOXO3a [44,45]. FOXOs can also cause cell cycle arrest at the G2/M checkpoint by inducing GADD45α [46,47], a protein that is important in DNA damage repair, genetic integrity maintenance, and cell survival. GADD45α interacts with the cdc25/cyclin B complex and the CDK inhibitor p21, promoting the initiation of DNA repair [46]. Thus, by modulating GADD45α, FOXO proteins participate in DNA conservation and repair, genomic integrity preservation, and reduction of oncogenic mutation accumulation [47]. FOXOs also protect cells from reactive oxygen species (ROS) through gene regulation. Indeed, FOXO3a is upregulated in response to ROS accumulation, leading to increased expression of its transcriptional targets involved in cell detoxification and cell survival, such as SOD2 and catalase [48]. Additionally, FOXO proteins regulate the expression of various genes participating in cell death pathways, including TRAIL [49], TRADD [50], and Bim [51]. In particular, treatment of chronic myelogenous leukemia cell lines driven by the BCR-ABL chimeric oncogene with a BCR-ABL inhibitor promoted the activation of FOXO3a-dependent Bim expression, resulting in apoptosis [52]. FOXO3a has been widely shown to act as a tumor suppressor not only in leukemia, but also in breast and prostate cancer [53]. Moreover, somatic deletion of multiple FOXO alleles in mice results in a cancer predisposition phenotype [54]. Interestingly, the longevity-related function of DAF-16 in *C. elegans* [55] is evolutionarily conserved. In recent years, the relationship between the FOXO3a genotype and human longevity has been extensively investigated. Numerous single nucleotide polymorphisms (SNPs) located in intronic regions of the gene have been shown to be associated with longevity in different human populations [56,57,58,59,60]. Notably, in humans the rs2802292 SNP G-allele at FOXO3a locus correlates with reduced frequency of age-related diseases in centenarians [61,62] and is associated with mortality risk reduction for coronary heart disease [63,64]. Moreover, it has been found to have enhancer functions, creating a binding site for HSF1 in response to stress stimuli [62]. FOXO proteins are also involved in cellular differentiation. In mouse models, the absence of FOXO3a induces deficiency in the ability of hematopoietic stem cells (HSCs) to repopulate the bone marrow, suggesting its potential role in the promotion and maintenance of undifferentiated HSCs [65].

More recently, new findings highlighted the importance of microRNAs (miRNAs) in the regulation of FOXO3a, affecting its impact on metabolism, stress response, cell cycle progression, cell proliferation, autophagy, and apoptosis [66,67,68]. FOXO3a mRNA is directly targeted by the miR-132/miR-212 cluster, with miR-132 regulating HSC cycling and function through FOXO3a. Notably, miR-132 is upregulated with age in HSCs [69]. This evidence is of particular interest, since various polymorphisms in the FOXO3a locus have been shown to be associated with human longevity [70]. A number of miRNAs directly targeting FOXO mRNAs are also implicated in tumor promotion, growth, and metastasis: for example, FOXO3a mRNA was found to be targeted by miR-182 in lung cancer [71] and melanoma [72]; likewise, it was inhibited by miR-155 in pancreatic cancer, where it resulted in oxidative stress [73]. Moreover, overexpressing miR-59272 and miR-13077 in primary prostate epithelial cell lines inhibited FOXO3a protein levels and increased cell proliferation, whereas suppressing their expression reversed these effects. Consistently, miR-592 levels were found to be elevated in CRC tissues and cells, leading to decreased FOXO3a mRNA and protein amounts [74], while overexpression of miR-551b in isolated primary ovarian cancer (OVCa) cells promoted proliferation, invasion, and chemoresistance of OVCa stem cells via the repression of FOXO3a and TRIM31 proteins [75]. 

FOXOs’ transcriptional activity and protein-protein interactions are regulated by a complex cascade of post-translational modifications (PTMs), including phosphorylation, acetylation, and ubiquitination. These modifications, known as the “FOXO code” [76], are the result of the integration of cellular stimuli acting on these transcription factors. Notably, they determine the cellular localization and activity of FOXO proteins, since nuclear FOXOs can exert their typical transcriptional regulatory activity, while cytoplasmic FOXOs are inactive and undergo proteasomal degradation. In addition, PTMs can promote FOXO3a translocation into the mitochondria [31]. As a result, FOXO PTMs influence cell fate. 

### 2.1. Phosphorylation of FOXO3a 

FOXO3a positive and negative regulation mainly involves phosphorylation events, which are mostly related to evolutionarily conserved signaling pathways sensing metabolic and oxidative conditions of the cell. FOXO3a main negative regulatory cascade is the insulin signaling pathway, which involves PI3K and AKT [77,78]. Furthermore, two additional oncogenic kinases, IKKβ and ERK, also contribute to the regulation of FOXO3a nuclear localization and activity, supporting its critical role in cancer cell survival. Activation of PI3K is induced by IGF-1 binding to its receptor IGF-1R. Activated PI3K phosphorylates the phosphatidylinositol-4,5-biphosphate (PIP2) membrane lipid to phosphatidylinositol-3,4,5-triphosphate (PIP3), which recruits AKT and PDK1 to the plasma membrane. This allows PDK1 to bind to and activate AKT, leading to the phosphorylation of numerous substrates, including threonine 32 and serine 253 on FOXO3a [37]. As a result, FOXO3a interacts with the chaperone protein 14-3-3. This event blocks FOXO3a nuclear import by interfering with its NLS region in the FHRE domain and shifts its localization to the cytoplasm, leading to FOXO3a activity inhibition [79]. Similar to AKT, the SGK kinase is also activated by PI3K and phosphorylates FOXO3a on the same residues [80]. IKKβ phosphorylates FOXO3a on S644, leading to its nuclear exclusion and promoting its degradation by a ubiquitin-proteasome-dependent pathway [33,37,81,82]. Involvement of the RAS-ERK axis, one of the main factors modulating FOXO3a activity in differentiation, proliferation and tumor progression, relies on ERK and FOXO3a direct interaction and on the resulting phosphorylation of FOXO3a S294, S344 and S425 residues. This PTM promotes FOXO3a nuclear exclusion [83]. As increased FOXO3a cytoplasmic relocalization enhances its susceptibility to degradation, this results in increased cell proliferation and tumorigenesis. 

In response to oxidative stress, FOXO3a is phosphorylated by kinases promoting its nuclear accumulation. This is the case for the upstream activator of the mitogen-activated protein kinase (MAPK) pathway, MST-1, which can phosphorylate FOXO3a, thereby disrupting its interaction with 14-3-3 and promoting its nuclear translocation [84]. Similarly, JNK-mediated phosphorylation upon oxidative stress results in FOXO3a nuclear localization and induction of its transcriptional activity [67]. JNK can also modulate FOXO3a activity indirectly by repressing the PI3K/AKT pathway [85,86]. A similar mechanism involves the MAPK p38, which promotes FOXO3a phosphorylation at S7 and its relocalization to the nucleus in response to chemotherapy [86]. Similarly, upon metabolic stress AMPK mediates FOXO3a phosphorylation, influencing its nucleo-cytoplasmic shuttling and transcriptional activity. AMPK has been shown to phosphorylate FOXO3a on at least six specific residues, but experimental evidence suggests that other sites may also be involved [87]. Moreover, under metabolic stress AMPK indirectly modulates FOXO3a transcriptional activity by activating SIRT1 [88,89]. AMPK can also be triggered by mitochondrial ROS through an alternative pathway that involves FOXO3a and its target genes SOD, catalase and PGC1α [90,91]. Remarkably, phosphorylation by AMPK is required for FOXO3a mitochondrial translocation in normal cells [92]. 

### 2.2. Acetylation/Deacetylation of FOXO3a 

Acetylation/deacetylation events are another important PTM that regulates FOXO3a transcriptional activity, especially under oxidative stress. This mechanism is evolutionarily conserved, since in *C. elegans* DAF-16 is positively regulated by acetylation [93,94]. FOXO3a acetylation is mediated by the acetyltransferases p300 and CBP, while deacetylation is catalyzed by NAD+-dependent deacetylases such as SIRT1 and SIRT3 [76,89,95,96]. FOXO3a acetylation/deacetylation status influences the activation of different transcriptional programs, with acetylation promoting apoptosis by FOXO3a transactivation of pro-apoptotic genes such as Bim, p21 and FASL6 [89]. Conversely, FOXO3a deacetylation by SIRT1 results in cell survival and suppression of FOXO3a-dependent apoptosis through transactivation of downstream targets involved in cell cycle arrest, DNA repair and antioxidant activity, such as GADD45α, SOD, and p27 [89]. Therefore, SIRT1-mediated FOXO3a deacetylation can be considered as a cell-dependent cytoprotective mechanism [37]. It has been found that SIRT3, the main mitochondrial sirtuin, can also efficiently deacetylate FOXO3a [97]. SIRT3 interacts directly with FOXO3a both in the mitochondria and in the nucleus. Into the mitochondria, their interaction promotes transcription of oxidative stress response genes such as catalase and SOD2 [92,98,99], while into the nucleus it enhances transcription of its nuclear target genes [100]. These events modulate FOXO3a ability to regulate cellular ROS accumulation. In mice, SIRT3-FOXO3a interaction improves cardiac hypertrophy by ROS modulation through induction of FOXO3a nuclear localization and activation of antioxidant gene transcription [100,101]. 

### 2.3. Ubiquitination of FOXO3a

FOXO3a protein levels and activity can also be regulated by ubiquitin-dependent protein degradation [102]. Ubiquitination can occur as mono-ubiquitination, which is reversible, or as poly-ubiquitination, which is not reversible and is mediated by the E3 ligases SKP2 and MDM2, resulting in proteasome-mediated degradation. MDM2 can induce both FOXO mono-ubiquitination and poly-ubiquitination, the latter being related to AKT phosphorylation of FOXO3a on serine 253. Conversely, ERK-phosphorylation induces FOXO3a mono-ubiquitination by MDM2 [83]. IKK phosphorylation of FOXO3a induces its proteasomal degradation. Since IKK expression promotes cancer cell proliferation and tumorigenesis, FOXO3a phosphorylation by IKK might represent a tumorigenesis-promoting mechanism [81].

### 2.4. Methylation of FOXO3a 

Lysine methylation of FOXO3a is not well characterized. It has been reported that the methyl-transferase SET9 directly methylates FOXO3a in vitro and *in cellulo* on lysine 271. Methylation decreases FOXO3a stability; however, it can increase FOXO3a transcriptional activity upon stress stimuli [103]. 

## 3. FOXO3a: A Cancer Therapy Opportunity

Worldwide, current cancer treatments are only 50% successful [104]. In fact, various resistance mechanisms interfere with drug effects and/or desensitize cells against death signals. Cancer cells can develop chemoresistance in the course of therapy or can exhibit innate resistance. Loss of FOXO function decreases the ability to induce cell cycle arrest and to repair damaged DNA, thereby leading to genomic instability and tumor development. Moreover, since the ability to induce apoptosis is compromised in the absence of functional FOXOs, abnormal cells succeed to survive, resulting in tumor expansion. As mentioned above, simultaneous genetic deletion of five FOXO alleles, corresponding to somatic FOXO1, FOXO3a, or FOXO4 in mice, resulted in a cancer-prone phenotype [54]. Further deletion of all FOXO alleles induced progressive tumors late in life, such as thymic lymphomas and hemangiomas, indicating that FOXOs act as putative tumor suppressors. Furthermore, FOXO inactivation at the protein and mRNA levels occurs in cancer cells via different oncogenic signaling pathways. FOXO3a tumor suppressor function was recognized in human breast cancer tissue samples, since its absence correlated with poor patient survival [81]. Additionally, low levels of FOXO3a protein expression are associated with poor prognosis in several types of cancers, including ovarian cancer, hepatocellular carcinoma, gastric cancer, and lung adenocarcinoma [105,106,107,108,109,110]. During breast tumor development, inhibition of FOXO3a transcriptional activity promotes cell transformation, tumor progression, and angiogenesis, while its overexpression inhibits tumor growth [37,81,111]. In several types of human cancer, FOXO3a downregulation results from post-translational regulation by kinases such as AKT, IKKβ, and ERK, as well as from the deregulation of upstream regulatory pathways such as PTEN. 

Based on their tumor suppressor activity, FOXO factors have been investigated as therapeutic targets in various cancers, especially as mediators of the cytostatic and cytotoxic effects of chemotherapeutic agents. Different strategies have been implemented to activate FOXO3a. As an example, paclitaxel–a chemotherapeutic agent used in the treatment of breast carcinoma - activates FOXO3a by reducing the activity of its upstream kinase AKT [85], thereby impairing the interaction between FOXO3a and the 14-3-3 protein [102] and thus 14-3-3-mediated FOXO3a nuclear export. Paclitaxel can also modulate FOXO3a activation through JNK, which promotes FOXO3a-DNA binding and activation of FOXO3a transcriptional program [67,85]. Doxorubicin treatment induces p38-mediated FOXO3a nuclear relocalization [86] in MCF breast carcinoma cells. On the other hand, doxorubicin leads FOXO3a to induce expression of the multidrug resistance gene ABCB1 in K562 doxorubicin-sensitive leukemic cells [112]. Another strategy for targeting FOXO3a in cancer therapy is based on the modulation of the FOXO3a-FOXM1 transcriptional axis. FOXM1 is a proto-oncogene acting as a transcriptional activator, which is involved in cell proliferation by regulating the expression of cell cycle genes like cyclin B1 and cyclin D1. Its upregulation is frequent in several human malignancies, including liver, breast, lung, prostate, uterus, colon, and pancreas cancer. Furthermore, FOXO3a directly represses the activity of FOXM1 [113,114]. Various anticancer drugs, including the “tinibs” agents (lapatinib, imatinib, and gefitinib), paclitaxel, cisplatin, and doxorubicin, can be used to modulate the FOXO3a-FOXM1 axis. Intriguingly, their cytostatic and cytotoxic effects are mediated by the activation of FOXO3a and/or the inhibition of FOXM1 [86,114,115,116,117]. Lapatinib, imatinib, and gefitinib interfere with the PI3K-AKT-FOXO3a-FOXM1 axis by blocking receptor tyrosine kinase autophosphorylation and downstream signaling. On the other hand, paclitaxel ability to trigger JNK leads to FOXO3a nuclear accumulation and, consequently, to FOXM1 inhibition. Similarly, doxorubicin promotes FOXO3a nuclear translocation by phosphorylation of the p38 MAPK; moreover, it can directly downregulate FOXM1 expression, similar to cisplatin and epirubicin [86,114,115,116,117].

In cancer cells in which the activity of sirtuins (i.e. SIRT4, SIRT5, SIRT6, and SIRT7) is upregulated, combined treatment with sirtuin inhibitors—such as sirtinol, salermide or EX527—and doxorubicin or epirubicin helps cells to overcome chemoresistance by mediating FOXO3a deacetylation and inhibition [114]. An interesting therapeutic opportunity is the combination of FOXO3a tumor suppressor activity regulators and other therapeutic agents to sensitize resistant tumor cells. In CRC cells, FOXO3a is a mediator of cisplatin cytotoxic effect. Inhibition of FOXO3a dephosphorylation and nuclear translocation induced by cisplatin by acting on the PI3K/AKT/FOXO3a axis causes CRC chemoresistance to the drug [116]. Similarly, activation of the PI3K/AKT/FOXO3a pathway plays an important role in 5-fluorouracil (5-FU) resistance in CRC. Co-treatment of CRC cells with 5-FU and the PI3K/mTOR inhibitor NVP-BEZ235 induces inhibition of the AKT survival pathway and activation of FOXO3a-mediated transcription of target genes related to cell death [118]. Additionally, the p38-FOXO3a axis can be targeted to overcome chemoresistance of CRC cells to chemotherapeutics. In vitro co-treatment with a p38 inhibitor, SB202190, and cisplatin induces FOXO3a activation and apoptosis mediated by FOXO3a target genes. Moreover, in vivo the same co-treatment results in tumor regression in a xenograft mouse model [119]. In estrogen receptor-negative breast cancer, activation of FOXO3a through inhibition of the PDK-1/AKT pathway results in sensitization of chemoresistant cells to tamoxifen [120]. Another combined therapy approach is based on EGFR/HER2 blocking agents, which are currently employed in clinical trials against breast, prostate, kidney, ovarian and lung cancer [121]. In particular, inhibition of the EGFR family by monoclonal antibodies such as trastuzumab or cetuximab impairs the PI3K pathway and promotes FOXO3a activity [122], while reconstitution of active FOXO3a results in sensitization of resistant cancer cells to EGFR/HER2 inhibitors like lapatinib and gefitinib [121]. Additionally, in an osteosarcoma model, FOXO3a activation induces Bim and apoptosis in cells exposed to ionizing radiation [123]. 

## 4. FOXO3a at the Interface Between Cell Death and Survival 

Depending on the cellular context, proteins generally considered as genuine tumor suppressors are sometimes required to be functional for full malignant phenotype acquisition as part of cancer energy metabolism reprogramming. This is the case for FOXO3a [91,124,125]. Indeed, further experimental evidence suggests a dual role for FOXO3a at the crossroad between cell survival and death. Recently, the effects of inducible FOXO3a activation or loss during tumor progression have been described in metastatic breast cancer. Noticeably, either modification of FOXO3a expression suppressed tumor growth and delayed metastasis [126]. Moreover, several studies performed in the last decade demonstrated that FOXO3a also has an unexpected function in the promotion of cancer cell survival, tumor expansion and metastasis, and that it modulates response to cancer treatment through a variety of mechanisms [127,128,129,130]. For instance, it plays a pro-invasion role in breast cancer by regulating the expression of matrix metalloproteinases (MMP) such as MMP-9 or MMP-13 [127,128]. FOXO3a pro-oncogenic functions have also been investigated in hepatocellular carcinoma (HCC) cells, where it activates serotonin-induced cell proliferation under serum deprivation conditions [129,130]. A more recent study revealed an association between FOXO3a overexpression and aggressive phenotypes with poor prognosis in HCC patients. Besides, downregulation of FOXO3a expression in HCC cell lines was shown to inhibit cell proliferation and migration. Similarly, FOXO3a protein expression has been associated with progression and poor prognosis in glioblastoma (GBM) patients. Indeed, FOXO3a overexpression considerably enhanced colony formation and invasion ability in GBM cells by activating c-Myc, MAP1LC3B and Beclin1 [131]. Consistently, FOXO3a knockdown strongly inhibited tumor progression. Other studies suggested that, at least in some cancer types including leukemia, activation of FOXO3a is initially required for apoptosis induction in response to chemotherapy, whereas its prolonged activity promotes drug resistance by increasing antioxidant defenses and DNA damage repair [112]. In this context, new treatments designed to inactivate FOXO3a might be good candidates to block tumor expansion and metastasis [127,128].

Not surprisingly, FOXO3a has further emerged as a major sensor for metabolic stress and chemotherapeutic drug response in cancer cells. In human colorectal and ovarian cancer, the AMPK/FOXO3a pathway acts as a functional metabolic switch capable of sensing variations in the AMP/ATP ratio. As a result, decreased glycolysis caused by inhibition of the p38α/HIF1α pathway activates FOXO3a transcriptional program in an AMPK-dependent manner [132]. Notably, p38α pharmacological inhibition is a promising strategy for tumors such as CRC, in which p38α is required for cell proliferation and survival [133,134]. For example, in CRC the combined use of SB202190, a selective p38 inhibitor, and sorafenib, a kinase inhibitor approved for the treatment of renal cell carcinoma and hepatocellular carcinoma, enhances the anti-cancer activity of sorafenib by inducing apoptosis [135]. In CRC, inhibition of the p38α signaling pathway significantly impairs intracellular levels of ATP and decreases HIF1α protein stability as well as the expression of key enzymes involved in aerobic glycolysis. The resulting metabolic stress activates AMPK, which induces FOXO3a nuclear accumulation, thereby promoting the transcription of its target genes related to autophagy, cell metabolism, cell cycle arrest, and cell death [134]. FOXO3a activation first leads to the transcription of genes involved in the autophagic flux, namely GABARAP, GABARAPL1, GABARAPL2, and MAP1LC3. Then, FOXO3a target genes involved in metabolism, such as PGC1α, PEPCK, and UCP2, are induced to convert in ATP the fatty acids and amino acids produced by the autophagic flux. Finally, FOXO3a promotes the upregulation of the cyclin D transcriptional repressor Bcl-6 and of the CDK inhibitors p21 and p27, which block the G1/S transition, leading the cell to exit from the cell cycle. If metabolic stress conditions persist, cells undergo cell death, which is mediated by the FOXO3a-dependent transcriptional activation of ATG6, ATG7, ATG12, and BH3-only proteins such as PUMA, Bim, BNIP3, and BNIP3L. The AMPK-dependent activation of FOXO3a transcriptional program in metabolic stress conditions causes tumor growth inhibition, both in vitro and in vivo [132], suggesting that pharmacological manipulation of the AMPK-FOXO3a axis is an interesting approach for cancer treatment. 

## 5. The Mitochondrial Arm of the AMPK-FOXO3a Axis 

Recently, a mitochondrial arm of the AMPK-FOXO3a axis has been uncovered. A growing body of evidence highlights FOXO3a involvement in various mitochondrial functions, such as ROS detoxification, mitochondrial fission, biogenesis, and morphology control [136,137]. For instance, the increase in mitochondrial ROS production required for HIF-1α stabilization upon hypoxia induces FOXO3a activation, which counteracts hypoxia-dependent ROS production and HIF-1α accumulation through modulation of c-Myc stability [136]. Recent studies have focused on AMPK ability to promote mitochondrial homeostasis by regulating mitochondrial fission, mitophagy, and transcriptional control of mitochondrial biogenesis under stress conditions [138]. Furthermore, the presence of AMPK substrates in the mitochondrial outer membrane, such as the mitochondrial fission factor and acetyl-CoA carboxylase 2, suggests that a pool of freely floating cytosolic AMPK can localize at least close to mitochondria in response to changes in energy status. In support of this hypothesis, it has recently been observed that a myristoylated form of AMPK is recruited to the mitochondria in response to mitochondrial damage to mediate autophagy-dependent mitochondrial removal [139]. Recent evidence suggests the existence of a mitochondrial pool of FOXO3a [92,98,140]. In mammalian cells (myotubes and fibroblasts), FOXO3a can accumulate into the mitochondria upon glucose restriction [92]. This event requires AMPK activation, which is regulated by nutrient conditions through the energy sensor pathway. Inside the organelles, FOXO3a physically interacts with mitochondrial RNA (mtRNA) polymerase and SIRT3, the main mitochondrial sirtuin [141]. Remarkably, SIRT3-FOXO3a mitochondrial interaction allows the formation of a FOXO3a-SIRT3-mtRNA polymerase complex, which binds to mitochondrial DNA (mtDNA) and activates the transcription of mitochondrial-encoded catalytic subunits of the oxidative phosphorylation machinery [92,98]. Therefore, this event results in increased respiration and supports cell energy metabolism. Importantly, SIRT3 activity is required for FOXO3a recruitment on FHRE sites in the D-loop regulatory region and for mtDNA binding, but is not necessary for FOXO3a mitochondrial localization. The newly identified mitochondrial arm of the AMPK-FOXO3a axis operates as a recovery mechanism to sustain cellular metabolism upon nutrient shortage in normal cells and can modulate the balance between oxidative phosphorylation and glycolysis in response to metabolic stress [92]. 

**Figure 1:**
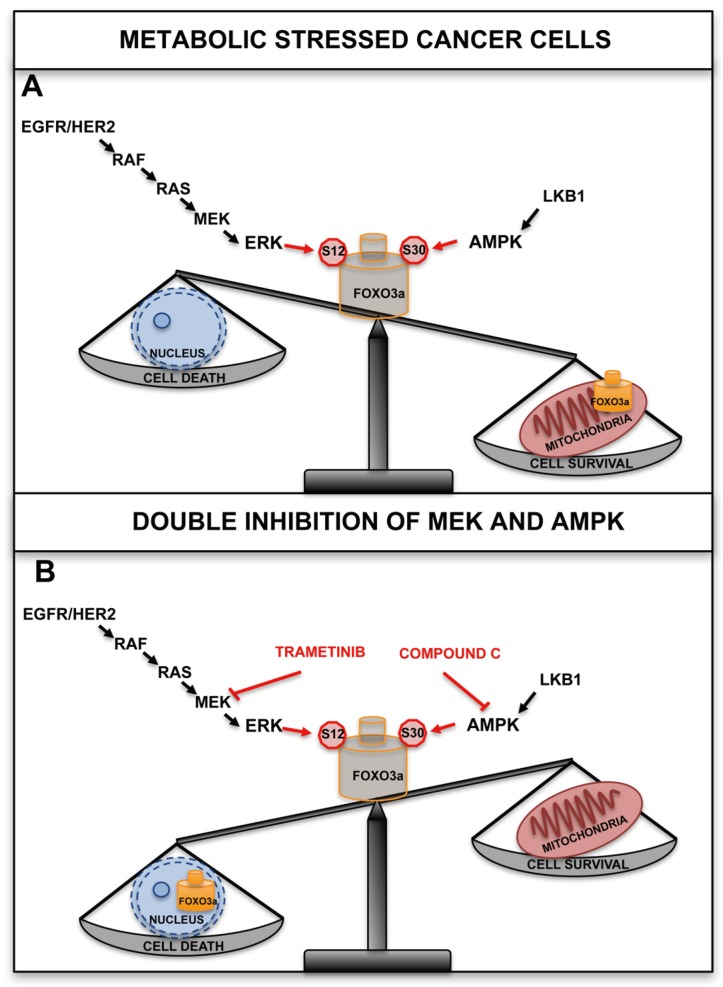
FOXO3a localizes to the mitochondria in tumor cells and tissues subjected to metabolic stress. (**A**) Two serine residues, S12 and S30, have been identified that are phosphorylated by the MEK/ERK and AMPK pathways, respectively, in response to metabolic stress. This leads to FOXO3a translocation into the mitochondria, where it binds to mitochondrial DNA (mtDNA) together with TFAM, mitochondrial RNA (mtRNA) polymerase, and SIRT3, and activates the expression of the mitochondrial genome with the final effect of sustaining the healthy and functionally active state of mitochondria in metabolically stressed cancer cells. (**B**) Trametinib (a MEK inhibitor approved for clinical use by the Food and Drug Administration (FDA) and compound C (an AMPK inhibitor) showed a synergistic cytotoxic effect in metabolically stressed cancer cells.

Recently, it has been shown that FOXO3a localizes to the mitochondria in tumor cells and tissues subjected to metabolic stress and cancer therapeutics, and the involved signaling pathways and molecular mechanisms have been characterized. The results indicate that similar to what happens for other nuclear-encoded mitochondrial proteins, the N-terminal domain of FOXO3a (amino acids 1-148) is required for proper recruitment to the mitochondria. Furthermore, the region encompassing residues 98-108, which contains overlapping consensus motifs for MPP and MIP mitochondrial peptidases, was found to be necessary for FOXO3a cleavage and import into the mitochondrial matrix. It is worth noting that this region is specific to FOXO3a (it is not conserved in other human FOXO members), and is evolutionarily conserved across species, showing a certain degree of similarity also in *C. elegans*. This study describes for the first time the existence of a cleaved form of FOXO3a, which loses most residues encompassing the N-terminal domain; however, as it retains an intact DBD (amino acids 149–242), it can efficiently bind to mtDNA and activate the expression of the mitochondrial genome. Two serine residues, S12 and S30, have been identified that are phosphorylated by the MEK/ERK and AMPK pathways, respectively, in response to metabolic stress [31]. This leads to FOXO3a translocation into the mitochondria, where it binds to mtDNA together with TFAM, mtRNA polymerase and SIRT3, and activates the expression of the mitochondrial genome with the final effect of sustaining the healthy and functionally active state of mitochondria in metabolically stressed cancer cells (Figure 1A, B). 

**Figure 2:**
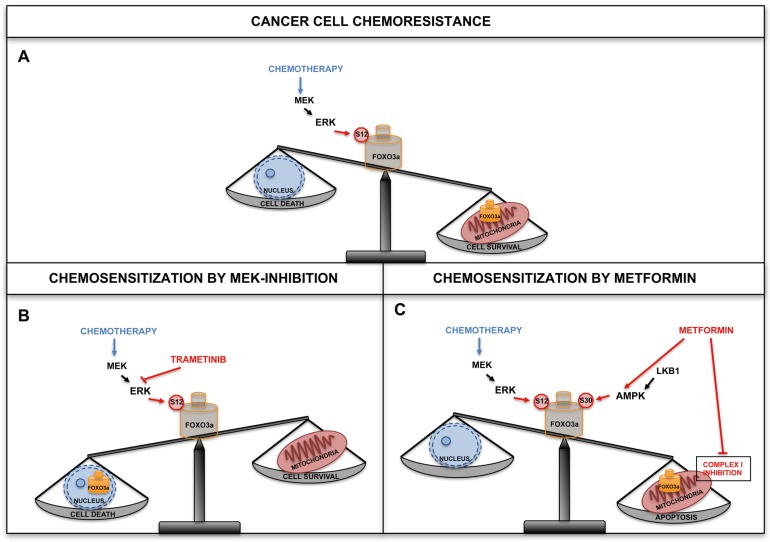
Combination therapy with chemotherapeutic agents and inhibitors of cancer-related pathways are predicted to overcome resistance mechanisms. (**A**) In cancer cells treated with chemotherapeutic agents, accumulation of FOXO3a into the mitochondria promoted chemotherapy resistance and cell survival in a MEK/ERK-dependent manner. (**B**) Combined therapy with MEK inhibitors and chemotherapeutic drugs is predicted to overcome resistance mechanisms and potentiate the antitumor activity of each agent. MEK inhibition by trametinib enhanced cell death when combined with chemotherapeutic agents in colorectal cancer (CRC) cells. (**C)** Metformin activity was mediated by AMPK and required mitochondrial FOXO3a (mtFOXO3a) in order to elicit a pro-apoptotic response in tumor cells. Indeed, a synergistic cytotoxic effect was observed when metformin was combined with chemotherapeutic agents.

Notably, both serine residues have been found to be phosphorylated in human samples of different types of cancer, thus identifying predictive phosphorylation signatures [142,143,144,145,146,147]. AMPK can directly phosphorylate FOXO3a at S30 of the N-terminal domain and this PTM is required for FOXO3a recruitment at the mitochondrial surface prior to undergoing translocation and processing. Importantly, S30 is evolutionarily conserved from *D. melanogaster* to humans and is part of a highly conserved subdomain that is shared by other human FOXOs. It has been previously shown that ERK can phosphorylate FOXO3a (at S294, S344 and S425), resulting in FOXO3a nuclear exclusion and increased proliferation in cancer cells [83]. The same study also revealed that ERK can specifically phosphorylate S12 in metabolically stressed cancer cells, and that this PTM is required for FOXO3a mitochondrial import. Importantly, S12 is less conserved across species than S30 and is not present in the other three members of the human FOXO family. In line with previous findings [92], the authors observed that in normal cells and tissues subjected to metabolic stress, S30 phosphorylation by AMPK was the only signal required to promote FOXO3a mitochondrial relocalization. This uncovers a distinctive feature compared to tumor cells, in which S12 phosphorylation by ERK was also needed for FOXO3a translocation into the mitochondria. This difference could be taken advantage of to devise targeted therapeutic interventions. Indeed, when cancer cells were treated with chemotherapeutic agents that are currently administered to CRC patients and whose activity has been shown to involve FOXO3a in cellular models, such as cisplatin, irinotecan, 5-FU and etoposide [91,109,117,118,119], mitochondrial FOXO3a (mtFOXO3a) was required for mitochondrial function preservation, chemotherapy resistance and cell survival in a MEK/ERK-dependent manner (Figure 2). 

Indeed, chemotherapeutic agents do not activate AMPK, and their activity do not require a phosphorylatable serine in position 30, with cell survival only relying on MEK-dependent ERK phosphorylation of S12. These findings were corroborated by data showing that MEK inhibition by trametinib enhanced cell death when combined with chemotherapeutics and synergized with irinotecan in inducing cytotoxicity in CRC cells (Figure 2B). Thus, when used in combination with chemotherapeutic drugs, MEK inhibitors seem to potentiate the antitumor activity of either single agent alone, allowing to overcome resistance. This is of particular significance, since phase II/III trials based on these strategies are already being conducted [148]. 

The analysis of FOXO3a mutant cells also revealed that the AMPK-mtFOXO3a axis is required for metformin to extensively induce apoptosis in cancer cells. Metformin is an antihyperglycemic agent commonly used in the treatment of type II diabetes and has been associated to diminished tumorigenesis in diabetic patients [149,150]. In a doxorubicin-resistant breast cancer cell line, metformin impaired mitochondrial membrane potential. In CRC cell lines, strong mitochondrial depolarization coincided with increased ROS production, which contributed to metformin antiproliferative effects. In glucose deprivation conditions, metformin sensitized cancer cells by inhibiting mitochondrial complex I, reducing the accumulation of unfolded proteins in the endoplasmic reticulum and promoting the activation of SIRT3. This enhanced metformin-initiated apoptosis, energetic stress and mitochondrial dysfunction, and induced AMPK overexpression [151,152,153]. Indeed, metformin activity was mediated by AMPK and required mtFOXO3a in order to elicit its pro-apoptotic effect in tumor cells (Figure 2C). It can be speculated that metformin created a vicious circle causing an imbalance in mitochondrial metabolism, which was sustained by mtFOXO3a but repressed by complex I inhibition by metformin itself. This could paradoxically be useful to circumvent mtFOXO3a-dependent chemoresistance and sensitize cancer cells to chemotherapy. Indeed, a synergistic cytotoxic effect has been observed when metformin was combined with irinotecan [31]. Notably, phase II/III clinical trials are currently evaluating the effect of metformin in combination with chemotherapeutic drugs as well as its cancer chemoprevention activity as a single agent [149,150]. Notably, metformin is capable of specifically targeting cancer stem cells, which play a crucial role in chemoresistance, through several signaling pathways including AKT/PI3K/mTOR, insulin/IGF1, MAPK, Sonic hedgehog, Wnt, TGFβ, Notch and NFKB [151]. 

## 6. Conclusion

In order to fully exploit the specificity of new targeted drug combinations, cancer therapies need to be tailored to each patient’s cellular circuitry, and clinical protocols should be designed based on each tumor’s molecular signature. However, high inter-tumor heterogeneity and chemoresistance are major obstacles to effectively developing and applying targeted therapeutic agents. In this context, detecting alterations across tumor genomes is expected to produce significant improvements in precision cancer medicine. 

Transcription factor dysregulation has been found to be a common phenomenon in human malignancies [154] and can lead to significant modifications in the expression of genes involved in complex biological processes. These changes are key determinants of tumor behavior as they contribute to cell proliferation, migration and metastasis, as well as to chemoresistance. Overall, targeting cancer-related pathways can influence transcription factor activity by modulating their cellular localization, protein/protein interactions and affinity to DNA binding sites. The development of CRC resistance to anticancer drugs depends on genomic alterations and pathogenic activation of various pathways that converge on the FOXO3a transcription factor. Hence, FOXO3a is a promising target for CRC treatment. Several existing therapeutic strategies modulate FOXO3a activity. In particular, pharmacological manipulation of FOXO3a subcellular localization could prove an effective approach (Figure 1). Indeed, in cancer cells treated with chemotherapeutic agents currently administered to CRC patients, accumulation of FOXO3a into the mitochondria has been found to promote chemoresistance and survival in a MEK/ERK-dependent manner (Figure 2). Recent reports further suggest that cancer cells can be sensitized to chemotherapeutics by inhibiting mitochondrial translation, since they are extremely sensitive to the disruption of oxidative phosphorylation. Importantly, combination therapy with chemotherapeutic agents and inhibitors of cancer-related pathways are predicted to overcome resistance mechanisms and potentiate the antitumor activity of each single agent. On the other hand, apoptosis induction by metformin in CRC has been shown to be mediated by AMPK and to require mtFOXO3a to elicit its pro-apoptotic and chemosensitization effect (Figure 2C). In the future, it will be crucial to establish whether, in addition to the MEK/ERK and AMPK cascades, FOXO3a N-terminus is targeted by other signaling pathways that can modulate its mitochondrial localization and function, and to determine the relevant triggering external stimuli. Moreover, further studies are needed to corroborate whether adding metformin to a chemotherapeutic regimen is an effective strategy to enhance drug sensitivity.
